# Lenvatinib-Induced Acalculous Cholecystitis—An Often-Unrecognized Toxicity: A Case Series and Literature Review

**DOI:** 10.3390/curroncol33030167

**Published:** 2026-03-14

**Authors:** Christos Cortas, Chloe Symeonidou, Haris Charalambous

**Affiliations:** 1 German Oncology Center, 4108 Limassol, Cyprus; 2 Bank of Cyprus Oncology Center, 2006 Nicosia, Cyprus; chloe.symeonidou@bococ.org.cy

**Keywords:** Lenvatinib, cholecystitis, TKI, adverse effects, supportive care

## Abstract

Lenvatinib is an oral multi-kinase inhibitor which is used for the treatment of renal cell carcinoma, non-iodine avid differentiated thyroid cancer, hepatocellular carcinoma, and endometrial cancer. We present a series of three (3) patients who, whilst on treatment with Lenvatinib, developed symptoms and radiological findings of acalculous cholecystitis. A radiological review of another nineteen (19) Lenvatinib-treated patients in our center was undertaken, with another three (3) patients exhibiting radiological features of acalculous cholecystitis with gallbladder wall thickening and pericholecystic fluid collection but without abdominal pain or clinical symptoms of cholecystitis. A literature review is also presented of all previous publications of Lenvatinib-induced acalculous cholecystitis, including the results of two pharmacovigilance studies. This review provides evidence that Lenvatinib-induced acalculous cholecystitis is not as rare as initially thought from the Lenvatinib licensing studies; hence, this is an adverse event that merits more attention. Oncologists using Lenvatinib should be aware of this potential toxicity and be familiar with its management.

## Simple Summary

Lenvatinib is an oral oncology drug which is used for the treatment of several different cancer types such as kidney, uterine, liver, and thyroid cancer. We present a study of twenty-two (22) patients receiving Lenvatinib in our oncology center, in which three (3) patients developed both clinical and radiological features of cholecystitis (inflammation of the gallbladder) without the presence of gallstones, which is called acalculous cholecystitis. Another three (3) patients had radiological features of acalculous cholecystitis but without abdominal pain or other clinical symptoms of cholecystitis, which has also been described in the past. We finally undertook a review of similar Lenvatinib-induced acalculous cholecystitis studies published in the literature, and our findings suggest that this toxicity is not as rare as suggested by the Lenvatinib licensing studies, and that oncologists using Lenvatinib need to be made aware of this potential side effect and its management.

## 1. Case 1

**Figure 1:**
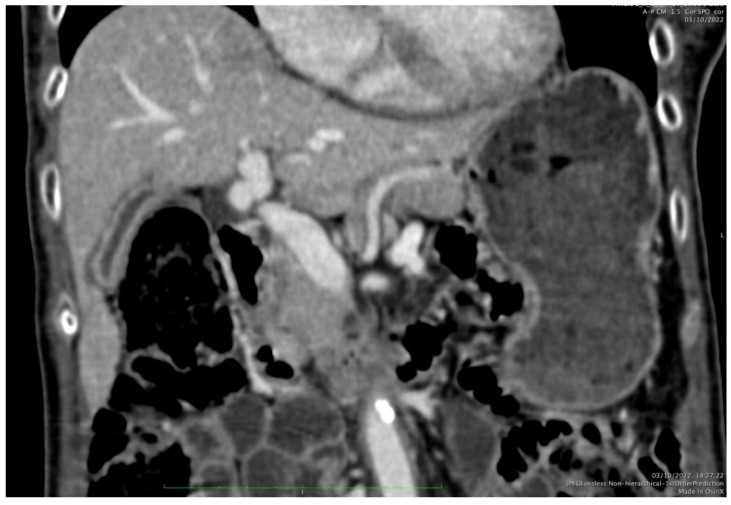
Abdominal CT with intravenous iodine contrast: peri-cystic fluid, inflammation, and wall thickening in a patient with abdominal pain while receiving Lenvatinib. Gallbladder stones were not identified.

A 72-year-old lady underwent total thyroidectomy in 2005 for a pT3 papillary thyroid cancer followed by radioactive iodine (RAI) treatment. She relapsed in 2016 with raised thyroglobulin but no radiologically clinically evident disease and underwent further treatment with RAI. In mid-2019, she had further disease recurrence with neck lymphadenopathy, which was treated with neck lymphadenectomy and further RAI. The iodine scan post RAI was negative, whilst a Positron Emission Tomography–Computerized Tomography (PET-CT) after treatment showed residual disease in cervical lymph nodes and lung metastases, and hence the patient was characterized as having RAI refractory disease. The patient was started on Lenvatinib 24 mg daily by an oncologist in Athens in December 2019 and returned to continue her treatment in Cyprus. Soon after, she developed nausea, anorexia, and pain in the right hypochondriac area, and following symptomatic treatment with anti-emetics and analgesia, a dose reduction to 20 mg was undertaken. Her symptoms failed to improve and in fact further deteriorated; hence, the Lenvatinib was stopped, and a Computerized Tomography (CT) scan was organized in March 2020, showing partial radiological response of her lung metastases, gallbladder inflammation, and a small amount of pericholecystic fluid without the presence of gallstones (Figure 1). At the same time, she had a mild elevation in transaminases, Aspartate Aminotransferase (AST) × 2 upper limit of normal (ULN), and a borderline rise in Alkaline Phosphatase (ALK) and gamma-glutamyl transferase (gGT) in March 2020, i.e., at 3 months from starting treatment. Baseline liver function tests were normal. These results subsequently improved on treatment discontinuation and initiation of lower-dose Lenvatinib. Drug discontinuation resulted in improvement in her symptoms. Subsequently, she restarted Lenvatinib at a lower dose of 14 mg Lenvatinib daily. However, a few weeks later, her symptoms re-appeared, especially abdominal pain and difficulty eating, resulting in a weight loss of approximately 5 kg. For this reason, treatment with Lenvatinib was again stopped. Further imaging continued to show radiological findings of acalculous cholecystitis. When her symptoms improved after Lenvatinib discontinuation, another attempt to restart Lenvatinib was made with a lower dose of 10 mg of Lenvatinib daily. Again, her symptoms re-appeared, leading to further discontinuation of Lenvatinib and further dose reduction to 8 mg, followed by 4 mg daily. On serial imaging, and on the lower dose of 4 mg, the radiology findings improved and almost completely normalized in October 2021. The patient remained on the lowest dose of 4 mg of Lenvatinib, with controlled thyroglobulin and no evidence of radiological disease progression and no abdominal pain. Even on the lower dose of Lenvatinib, she had difficulty tolerating it, and eventually, due to significant malaise, she was switched to sorafenib in June 2023 given the lack of progression on Lenvatinib. She remained on Lenvatinib treatment for forty (40) months. She continued on sorafenib until January 2025, when she was switched to cabozantinib due to disease progression, and finally passed away in April 2025.

## 2. Case 2

**Figure 2:**
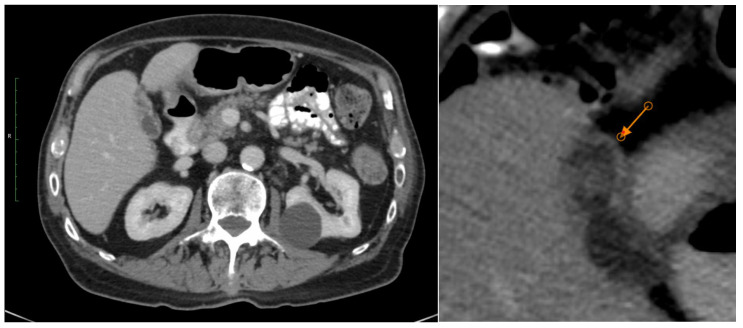
CT of abdomen with IV contrast: documentation of cholecystitis without evidence of stones or biliary tree obstruction.

A 73-year-old man underwent parotidectomy for adenoid cystic carcinoma of the parotid staged pT1N0 R1 in April 2015 and then he received adjuvant radiotherapy from May 2015 until July of the same year. In August 2017, he developed solitary lung metastasis with chest wall invasion and underwent surgical metastectomy in view of the long disease progression-free interval from his parotidectomy and the solitary nature of the relapse. Eighteen (18) months after the metastatectomy, in February 2019, he relapsed with a paraspinal soft tissue mass, which was treated with external beam radiotherapy. Finally, in February 2020, he suffered systemic relapse, with lung and bone metastases, which was treated with combination palliative chemotherapy with cisplatin, doxorubicin, and cyclophosphamide, to which he had a modest radiological response after three cycles of treatment. However, he suffered further disease progression at the time of completion of six cycles of chemotherapy. Given the systemic and symptomatic nature of his disease, he was prescribed Lenvatinib 24 mg daily in May 2020. About two (2) months later, he developed right-sided hypochondriac abdominal pain, associated with elevated inflammation markers (C-Reactive Protein (CRP) 199 mg/dL). He also had a mild increase in Alanine Aminotransferase (ALT) and lactate dehydrogenase (LDH), as well as total white blood cell count (WCC), with 84% neutrophilia. Those results subsequently improved on treatment discontinuation and initiation of a lower dose of Lenvatinib. However, in May 2021, there was evidence of a mild increase again in ALT and AST, which again was transient. The CT scan showed that he had mild radiological improvement, but according to the Response Evaluation Criteria in Solid Tumors (RECIST) criteria, he had stable disease and evidence of gallbladder distention without any possible identifiable cause of obstruction. Symptomatic management with analgesia was initiated and Lenvatinib was reduced to 20 mg. Due to persistence of the abdominal symptoms, despite use of analgesics, a further dose reduction to 14 mg was initiated one month later. Radiological findings of acalculous cholecystitis persisted on the CT scan, which was done in October 2020 (Figure 2). In December 2020, the patient complained again of right hypochondriac abdominal pain, and image findings of acalculous cholecystitis were observed on a CT scan. The Lenvatinib dose was reduced to 10 mg daily and symptoms improved. The patient subsequently continued to receive Lenvatinib at this dose without further abdominal pain until July of 2021, when he developed disease progression, and his treatment was changed again to palliative chemotherapy. He remained on Lenvatinib treatment for a period of fourteen (14) months. He subsequently received three (3) lines of palliative chemotherapy, including Cyclophosphamide/Doxorubicin/Cisplatin, Carboplatin/Paclitaxel, and Carboplatin/Vinorelbine, from July 2021 until February 2023. He finally passed away in April 2023.

## 3. Case 3

**Figure 3:**
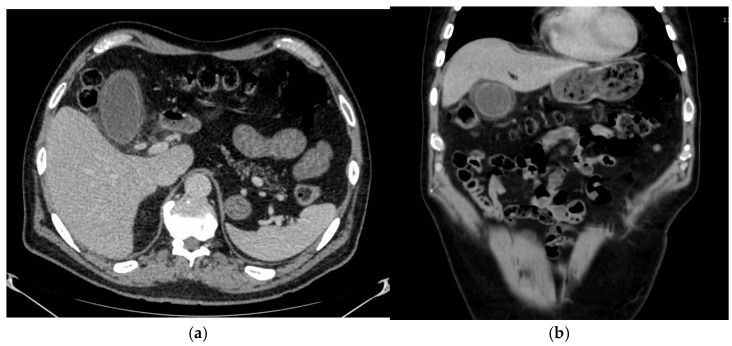
(**a**) horizontal plane view of a CT of abdomen with contrast: increased enhancement of the gallbladder wall with pericholecystic edema and fat stranding extending to common bile duct (**b**) transverse plane view of a CT of abdomen with contrast: increased enhancement of the gallbladder wall with pericholecystic edema and fat stranding extending to common bile duct and first part of duodenum.

A 53-year-old male patient was diagnosed with metastatic kidney cancer in July 2019. He underwent cytoreduction nephrectomy, followed by palliative radiotherapy for solitary sacral metastasis, and he then started on Pembrolizumab and Axitinib combination therapy. Following disease progression to this treatment in July 2021, he received cabozantinib until January 2022. He then started Lenvatinib 18 mg daily combined with Everolimus 5 mg daily in February 2022 as third-line treatment. A CT scan after three (3) months of treatment showed a good partial radiological response with improvement in his metastatic disease. In early July 2022, the patient presented with abdominal pain, and a CT scan was organized. Pericholecystic edema and fat stranding extending to the common bile duct were observed in the CT scan (Figure 3). There was no change in his liver function tests or any other blood parameters. Initially, Lenvatinib was stopped with improvement in his symptoms and then restarted at a lower dose of 14 mg, with good tolerance. Lenvatinib and Everolimus were discontinued after disease progression in November 2022. He received nine (9) months of Lenvatinib-based therapy. In December 2022, he was started on Sunitinib and finally passed away in July 2023.

## 4. Discussion

Lenvatinib is an oral multi-kinase inhibitor targeting Fibroblast Growth Factor Receptors (FGFRs) 1–4, platelet-derived growth factor receptor alpha (PDGFR)a, Rearranged during Transfection (RET), KIT proto-oncogene, receptor tyrosine kinase (KIT), and Vascular Endothelial Growth Factor (VEGF) 1, 2, and 3 receptor proteins [1]. Currently, Lenvatinib is indicated for the treatment of radioactive iodine refractory differentiated thyroid cancer (DTC), unresectable hepatocellular carcinoma, advanced renal cell carcinoma, and advanced endometrial cancer, as monotherapy or in combination with Everolimus or with Pembrolizumab [2,3,4,5,6]. Lenvatinib has a high rate of adverse effects of any grade, which reaches approximately 98% [6]. Grade 3 or 4 toxicity is seen in 49.6% of the cases and includes hypertension (44.4%), proteinuria (10.7%), diarrhea (9.2%), renal failure (3.1%), hepatic failure (5.4%), gastroenteric fistula (1.9%), and arterial and venous thromboembolic events (2.2%) [6].

Cholecystitis, while described as extremely rare in the SELECT study on metastatic thyroid cancer, occurring in 0.4% of patients [2], and as 0.2% for grade>/3 in the phase III study on hepatocellular carcinoma [3], is described as common in the Summary of the Product Characteristics (SPC) [6], with a 1–10% frequency. In our series, acalculous cholecystitis was diagnosed in three (3) out of 22 patients, i.e., in 13.6% of patients. All three (3) patients presented with abdominal pain and with radiological findings of acalculous cholecystitis. The symptoms were severe enough to warrant treatment interruption and dose reduction on multiple occasions, and for all patients, there was significant impact on their quality of life and ability to receive the drug. Dose reductions were made down to 10 mg in one patient and to 4 mg in another patient on monotherapy, with an initial starting dose of 24 mg. In the other patient on combination therapy with Everolimus and Lenvatinib, the dose reduction was from 18 mg to 14 mg. With great care and effort, all patients managed to continue the drug and have significant periods on treatment without disease progression.

A literature review was conducted on PubMed by searching the terms “Acalculous Cholecystitis” and “Lenvatinib”. The search was updated on 22nd July 2025 (“cholecystitis”[Title/Abstract]) AND (“lenvatinib”[Title/Abstract]). Five (5) papers were initially identified; these studies provided reference to another three (3) papers. Two (2) case reports relating to this [7,8], four (4) case series [9,10,11,12], and two (2) pharmacovigilance studies [13,14] have been reported regarding Lenvatinib and cholecystitis. In a single-center retrospective study by Kurokawa et al., looking at pathologic hepatobiliary findings, which the authors called pancreatobiliary inflammation (PBI), including symptomatic pancreatitis, cholecystitis, and cholangitis in patients receiving Lenvatinib, seven (7) patients out of 78 were found to have cholecystitis, four (4) of those without gallstones [9]. There were also two (2) patients with cholangitis, one (1) without gallstones, and one patient with pancreatitis. Note that in this study, the majority of patients, sixty-two (62), had hepatocellular cancer, and sixteen (16) had thyroid cancer. Finally, they found that Lenvatinib-induced pancreatobiliary inflammation (PBI) led to a significantly shorter treatment duration [9]. In another single-center case series by Di Stefano et al., the incidence of acalculous cholecystitis was even higher, since 5 out of 13 patients (38.4%) were found to have cholecystitis and needed reduction or short interruption, with one patient out of the five having a small 9 mm stone in the infundibulum of the gallbladder [10]. In another retrospective single-center case series examining the incidence of hepatobiliary adverse events in 36 patients treated with Lenvatinib for thyroid cancer, Nervo et al. describe five (5) patients (14.7%) with cholecystitis; however, four out of five patients also had gallstones [11]. Finally, Aydermili et al. presented real-world data from three Dutch medical centers from patients with metastatic thyroid cancer treated with Lenvatinib, and in this series, 3 out of 39 patients (8%) developed cholecystitis/gallstones; it is not clear from the publication if all cases were due to acalculous cholecystitis or if they were associated with gallstones [12]. All the reports of Lenvatinib-induced acalculous cholecystitis according to the literature can be found in Table 1.

Cholecystitis and cholangitis were examined in a pharmacovigilance study by Lu et al. [13], using the FDA Adverse Event Reporting System (FAERS). Compared to the full FAERS database examined in this study, patients receiving Lenvatinib had a 20 times greater chance of presenting with cholecystitis (95% CI 17.3–22.7). Lenvatinib was also found to have an increased incidence of cholecystitis compared to other anti-VEGF TKIs, with a relative risk (RR) of 4.47 (95% CI 4.61–6.62). Even when hepatobiliary and pancreatic cancers were excluded, Lenvatinib resulted in a higher risk of cholecystitis compared to other VEGFR TKIs with an RR = 6.6 (95% CI 5.4–8.2).

Beyond symptomatic presentation with abdominal pain, acalculous gallbladder inflammation or cholecystitis may have a pre-clinical phase, with radiological findings of gallbladder inflammation, but without clinical symptoms. We undertook a radiological review of all twenty (22) patients treated in our center with Lenvatinib to investigate this further. Hence, a radiology review of the remaining nineteen (19) “asymptomatic” patients, i.e., without abdominal pain, was conducted. Three (3) of these patients had radiological findings of acalculous cholecystitis, with gallbladder wall thickening and pericholecystic fluid collection. Another patient displayed gallbladder wall thickening during his treatment; however, the imaging findings were disease-related and not considered to be Lenvatinib-related. Additionally, three (3) patients had previously undergone cholecystectomy, and two (2) patients had imaging findings of gallbladder stones, which were also considered unrelated. Table 2 illustrates a detailed radiology review of all the patients in our center that received Lenvatinib.

Kurokowa et al. [9] described a radiological entity of asymptomatic gallbladder inflammation as Asymptomatic Gallbladder Subserosal Edema (AGSE), which they consider to be related to pancreatobiliary inflammation (PBI) and in their series was calculated to be approximately 13%. AGSE was described to improve after Lenvatinib dose reduction. The clinical importance of AGSE is not clear, and further studies may shed light on this issue. The radiology review from our center, with three out of nineteen patients (16%) having radiological evidence but not clinical evidence of acalculous cholecystitis, is in fact similar to the Kurokawa series.

It is also worth noting that in the Di Stefano series [10], two out of five patients diagnosed with cholecystitis had vomiting, anorexia and jaundice, and a rise in liver function tests, but not abdominal pain in the presence of typical radiological changes compatible with acalculous cholecystitis, hence suggesting a variation in the symptoms and presentation of acalculous cholecystitis, and hence series that did not consider cases without abdominal pain, as consistent with cholecystitis, may have under-reported and underestimated the incidence of this toxicity.

In Table 1, we include the management of this toxicity, where one can see the two different strategies employed in the published case series in the literature, namely surgical versus conservative management. Nervo et al. employed cholecystectomy in all five patients and then resumed Lenvatinib at full dose, whilst Ishigaki et al. performed gallbladder drainage, but the patient was not able to continue Lenvatinib, as on rechallenge the cholecystitis symptoms recurred. The alternative strategy is to avoid surgery and decrease the Lenvatinib dose, which was employed by all the other series, including ours and those by Kurokowa et al. and Di Stefano et al., as well as the case report by Honda et al. The fact that the symptoms improved in the majority of patients on reducing the dose would suggest a dose-related pathogenesis and associated risk of acalculous cholecystitis; hence, that acalculous cholecystitis may be dose-dependent on the Lenvatinib dose for most of the patients.

Three different pathophysiological mechanisms have been proposed regarding the pathogenesis of acalculous cholecystitis [7,13]. Firstly, inhibition of VEGFR signaling can result in platelet activation and arterial thromboembolism in the gallbladder, which culminates in ischemia and acalculous cholecystitis [15]. This is supported by the fact that Lenvatinib has a similar mechanism of action to other VEGFR TKIs, which also result in cholecystitis, as a result of microthrombi formation and ischemia [16]. Unexpected thromboembolic events have been well described from TKIs in the past, and this has been proposed as a possible mechanism in another case report [17]. Secondly, it has been proposed that as biliary tract cells express high levels of VEGFR 2 and 3, due to Lenvatinib-induced VEGFR blockade, there is disruption of proliferation of biliary tract cells and cholestasis, which results in inflammation and cholecystitis, or generally biliary disease [17]. Finally, it has been suggested that acalculous cholecystitis is a result of a general proinflammatory altered state due to the TKI, especially relevant to Lenvatinib, which has predominantly biliary excretion, resulting in its accumulation and that of metabolites in the biliary fluids and trunks [10,18].

Acknowledging the limitations of a retrospective study and the small number of cases from our series, we identified three cases with symptoms of abdominal pain and radiological features of acalculous cholecystitis, resulting in a 13.6% incidence. This, especially when compared with the data of Di Stefano et al. [10] showing a 38.4% incidence and those of Kurokawa et al. [9] showing a 6.4% incidence, without considering the studies by Nervo et al. [11] and Aydermili et al. [12] where patients with gallstones were also included, suggests that this toxicity may be under-reported in the literature, especially in the licensing studies, and may be at the higher end or exceed the 1–10% incidence quoted in the SPC. Furthermore, patients may not have abdominal pain and still have radiological changes compatible with gallbladder inflammation, e.g., the AGSE entity, and careful monitoring of these patients would be required. Finally, some patients without abdominal pain may have other symptoms, e.g., nausea, anorexia, and weight loss, in the presence of radiological features of cholecystitis, which would also need to be recognized; hence, awareness of this toxicity is necessary to be able to address these symptoms.

Oncologists may be unaware of acalculous cholecystitis as a potential toxicity of Lenvatinib and possibly attribute abdominal pain, nausea, or anorexia to other causes. Hence, in clinical practice, for patients receiving Lenvatinib, it is important if patients develop hypochondriac pain, nausea, anorexia, or a rise in liver enzymes that oncologists organize a CT or Ultrasound/Magnetic Resonance Imaging of the abdomen to investigate further, considering acalculous cholecystitis in the differential diagnosis. Once a radiological and clinical diagnosis is made, we suggest initially implementing conservative management, with interruption and dose reduction of Lenvatinib [19]. For patients that do not improve clinically or radiologically, and where symptoms are severe, therapeutic or even prophylactic cholecystectomy should be considered. In severe cases, cholecystectomy is justified to prevent complications such as gangrene of the gallbladder wall, empyema, formation of abscesses, or perforation, which can result in high mortality rates [20]. In our series, as well as in the study by Di Stefano et al. [10], no patients underwent cholecystectomy, but exactly the opposite occurred in the study by Nevro et al. [11], where all five patients who developed cholecystitis underwent cholecystectomy. Prior to cholecystectomy, Lenvatinib needs to be discontinued for at least 5–7 days, taking into account its half-life, to prevent problems with wound healing or increased bleeding related to its mode of action. Finally, beyond oncologists, radiologists also need to be alert to this toxicity as well as the AGSE entity.

## 5. Conclusions

We present three (3) cases of symptomatic acalculous cholecystitis from a single cancer center over a period of three (3) years in a cohort of twenty-two (22) patients receiving Lenvatinib, and a further three (3) patients with radiological features of acalculous cholecystitis but without abdominal pain. Oncologists using Lenvatinib should be aware of this potential toxicity and that right-sided hypochondriac pain, nausea, fever, and a rise in liver function tests may be due to acalculous cholecystitis, and they should investigate and manage patients on Lenvatinib accordingly.

## Figures and Tables

**Table 1:** Publications of Lenvatinib-induced acalculous cholecystitis.

** Authors **	** Year **	** Number of Cases **	** Cancer Type **	** Treatment **	** Outcome **
Honda et al. [7]	2020	1	HCC	Dose reduction, conservative treatment	Gallbladder perforation
Kurokawa et al. [8]	2021	4	HCC, RAI-DTC	Dose reduction	Unknown
Di Stefano et al. [9]	2021	5	RAI-DTC	Conservative treatment, dose reduction, UDCA	Symptom resolution and normalization
Nervo et al. [11] *	2020	5	RAI-DTC	Four cases of laparoscopic and one case of open cholecystecomy, treatment break, continue treatment with same dose	Symptom resolution, one case of delayed wound healing
Aydemirli et al. [12] **	2020	3	RAI-DTC	Unknown	Unknown
Ishigaki et al. [13]	2020	1	HCC	Treatment break, percutaneous gallbladder drainage	Recurrent cholecystitis when Lenvatinib was resumed, permanent treatment discontinuation

HCC: hepatocellular carcinoma, RAI-DTC: radioiodine refractory differentiated thyroid cancer, UDCA: Ursodeoxycholic Acid. * Imaging study revealed gallstones in four out of five cases; ** presence of gallstones is not reported in this study.

**Table 2:** Findings of radiology review the hepatobiliary tract of all patients who received Lenvatinib in our center.

	** Age **	** Symptomatic **	** Duration of Treatment **	** Diagnosis **	** Radiological Findings **
1	72	Yes	39 months	PTC	Pericholecystic fluid, inflammation and wall thickening
2	51	No	11 months	ACC of lung	-
3	68	Yes	15 months	ACC of parotid	Gallbladder wall enhancement and pericholecystic fluid
4	62	No	5 months	HCC	Gallstones
5	60	No	2 months	Endometrial cancer	-
6	73	No	15 months	Endometrial cancer	-
7	59	No	1 month	RCC	Gallbladder wall thickening (disease-related)
8	46	No	19 months	Mucoepidermoid of parotid	-
9	53	No	53 months	RCC	-
10	50	Yes	8 months	RCC	Enhancement of the gallbladder wall with pericholecystic edema and fat stranding
11	46	No	6 months	RCC	-
12	56	No	8 months	Endometrial cancer	Cholecystectomy
13	47	No	2 months	RCC	Hepatectomy, cholecystectomy
14	59	No	2 months	Endometrial cancer	-
15	61	No	36 months	ACC of salivary gland	Increased wall enhancement and mild pericholecystic stranding
16	56	No	13 months	RCC	Gallstones
17	60	No	3 months	Endometrial cancer	Cholecystectomy
18	76	No	14 months	RCC	-
19	75	No	4 months	Endometrial cancer	-
20	30	No	5 months	RCC	-
21	70	No	18 months	Endometrial cancer	Gallbladder enhancement and small cholecystic edema
22	75	No	2 months	Endometrial cancer	Cholecystectomy

PTC: papillary thyroid carcinoma, ACC: adenocystic carcinoma, HCC: hepatocellular carcinoma, RCC: renal cell carcinoma.

## Data Availability

Research data can be provided pseudonymously upon request to the authors and after receiving approval from the National Bioethics Committee.
